# Excess mortality in the first COVID pandemic peak: cross-sectional analyses of the impact of age, sex, ethnicity, household size, and long-term conditions in people of known SARS-CoV-2 status in England

**DOI:** 10.3399/bjgp20X713393

**Published:** 2020-10-20

**Authors:** Mark Joy, FD Richard Hobbs, Jamie Lopez Bernal, Julian Sherlock, Gayatri Amirthalingam, Dylan McGagh, Oluwafunmi Akinyemi, Rachel Byford, Gavin Dabrera, Jienchi Dorward, Joanna Ellis, Filipa Ferreira, Nicholas Jones, Jason Oke, Cecilia Okusi, Brian D Nicholson, Mary Ramsay, James P Sheppard, Mary Sinnathamby, Maria Zambon, Gary Howsam, John Williams, Simon de Lusignan

**Affiliations:** Nuffield professor of primary care and head of department;; Nuffield professor of primary care and head of department;; National Infection Service, Public Health England, London.; Nuffield Department of Primary Care Health Sciences, University of Oxford, Oxford.; National Infection Service, Public Health England, London.; Nuffield Department of Primary Care Health Sciences, University of Oxford, Oxford.; Nuffield Department of Primary Care Health Sciences, University of Oxford, Oxford.; Nuffield Department of Primary Care Health Sciences, University of Oxford, Oxford.; National Infection Service, Public Health England, London.; University of Oxford and honorary associate scientist, Centre for the AIDS Programme of Research in South Africa (CAPRISA), University of KwaZulu–Natal, Durban, KwaZulu-Natal, South Africa.; National Infection Service, Public Health England, London.; Nuffield Department of Primary Care Health Sciences, University of Oxford, Oxford.; Nuffield Department of Primary Care Health Sciences, University of Oxford, Oxford.; Nuffield Department of Primary Care Health Sciences, University of Oxford, Oxford.; Nuffield Department of Primary Care Health Sciences, University of Oxford, Oxford.; Nuffield Department of Primary Care Health Sciences, University of Oxford, Oxford.; National Infection Service, Public Health England, London.; Nuffield Department of Primary Care Health Sciences, University of Oxford, Oxford.; National Infection Service, Public Health England, London.; National Infection Service, Public Health England, London.; Royal College of General Practitioners, London.; Nuffield Department of Primary Care Health Sciences, University of Oxford, Oxford.; Nuffield Department of Primary Care Health Sciences, University of Oxford, Oxford.

**Keywords:** medical record systems, computerized, mortality, pandemics, sentinel surveillance, severe acute respiratory syndrome coronavirus 2

## Abstract

**Background:**

The SARS-CoV-2 pandemic has passed its first peak in Europe.

**Aim:**

To describe the mortality in England and its association with SARS-CoV-2 status and other demographic and risk factors.

**Design and setting:**

Cross-sectional analyses of people with known SARS-CoV-2 status in the Oxford RCGP Research and Surveillance Centre (RSC) sentinel network.

**Method:**

Pseudonymised, coded clinical data were uploaded from volunteer general practice members of this nationally representative network (*n* = 4 413 734). All-cause mortality was compared with national rates for 2019, using a relative survival model, reporting relative hazard ratios (RHR), and 95% confidence intervals (CI). A multivariable adjusted odds ratios (OR) analysis was conducted for those with known SARS-CoV-2 status (*n* = 56 628, 1.3%) including multiple imputation and inverse probability analysis, and a complete cases sensitivity analysis.

**Results:**

Mortality peaked in week 16. People living in households of ≥9 had a fivefold increase in relative mortality (RHR = 5.1, 95% CI = 4.87 to 5.31, *P*<0.0001). The ORs of mortality were 8.9 (95% CI = 6.7 to 11.8, *P*<0.0001) and 9.7 (95% CI = 7.1 to 13.2, *P*<0.0001) for virologically and clinically diagnosed cases respectively, using people with negative tests as reference. The adjusted mortality for the virologically confirmed group was 18.1% (95% CI = 17.6 to 18.7). Male sex, population density, black ethnicity (compared to white), and people with long-term conditions, including learning disability (OR = 1.96, 95% CI = 1.22 to 3.18, *P* = 0.0056) had higher odds of mortality.

**Conclusion:**

The first SARS-CoV-2 peak in England has been associated with excess mortality. Planning for subsequent peaks needs to better manage risk in males, those of black ethnicity, older people, people with learning disabilities, and people who live in multi-occupancy dwellings.

## INTRODUCTION

The severe acute respiratory distress syndrome coronavirus 2 (SARS-CoV-2) pandemic has passed its first peak in many countries in Europe, where the speed of implementing lockdown has predicted mortality.^1^ The UK has had one of the highest SARS-CoV-2 associated mortality rates in Europe with >42 000 deaths. The European mortality project (EUROMOMO) lists England as the only country with an ‘Extremely High Excess’, and substantially greater than that of the devolved nations Scotland, Wales, and Northern Ireland.^2^ The reasons for this difference, despite a unified public health response, are unclear.^3^ There has been concern about excess mortality in care homes,^4^ and that it may be indicative of widening social inequality.^5^ Furthermore, England is also among the most densely populated countries in the world with 430 people per square kilometre — the highest in Europe — and London is the fifth most densely populated city globally.^6^

Sentinel systems, such as Oxford RCGP Research and Surveillance Centre network (RSC), were primarily established to monitor influenza infections and vaccine effectiveness.^7^ Their data contribute to understanding of excess winter mortality, though this has generally been in the context of influenza vaccine effectiveness.^8^ The role of Oxford RCGP RSC has evolved to support SARS-CoV-2 surveillance during the pandemic.^9^ Across the whole sentinel network (*n* = 4 413 734) between 28 January and 4 April 2020, 3802 tests were recorded,^10^ with the number rising to >11 000 by the end of this study period. Establishing SARS-CoV-2 status has become progressively easier as more testing has become available. Issues of test availability in primary care have been compounded by the increasing use of remote consultation during lockdown in the UK. Results of SARS-CoV-2 tests may originate in hospital or the sentinel network, as symptomatic patients have bypassed primary care as their initial healthcare contact. Multiple changes in coding used to record SARS-CoV-2 status in computerised medical record (CMR) systems have necessitated the development of a unifying ontology.^11^

**Table:** How this fits in

The UK had one of the highest SARS-CoV-2 associated mortality rates, with >42 000 deaths during the first wave of infection. Concerns about excess mortality still exist in care homes and widening social inequality has been suggested as a possible associated factor. Published reports showing disparities in SARS-CoV-2 infection and its impact on ethnic and socioeconomic variables have not included data on household size or clinical risks. Results from this observational cohort study showed living in households of ≥9 occupants was associated with a fivefold increase in relative mortality in the general population. Among people with known SARS-CoV-2 status (clinical or virological diagnosis), male sex, population density, black ethnicity (compared to white), and people with long-term conditions or learning disabilities had a higher odds of mortality. These findings reinforce the importance of the need for risk reduction strategies to reduce ethnic disparities, the impact of large household size, and increased risk associated with long-term conditions and learning disability.

The aim of this study was to describe the rate of all-cause mortality throughout the first peak of SARS-CoV-2 as recorded in the Oxford RCGP RSC; the impact of age, sex, and household size on any excess mortality observed; and the association of SARS-CoV-2 status and demographic and clinical risks factors with mortality.

## METHOD

### Study overview

This study used an observational cohort study design. Three main analyses are presented. First, the peak in mortality associated with the first SARS-CoV-2 peak is reported. The sentinel network mortality in the Oxford RCGP Research Surveillance Centre in 2020 is compared with 2019 for the same weeks reported by the UK Office of National Statistics (ONS).

Second, a relative survival analysis is conducted, comparing the mortality for 2020 with 2019, and estimating excess mortality across the whole population using ONS mortality rates for 2019. Finally, the association between SARS-CoV-2 status, demographic and clinical risk factors, and mortality is explored. Odds ratios (ORs) are estimated for all-cause mortality, and the modifying effect of age, sex, SARS-CoV-2 status, and household size examined. Models were further adjusted for ethnicity, socioeconomic status, smoking status, and underlying health conditions. The study data were collected from weeks 2–20 of 2020.

### Setting and participants

The study population included all patients registered at general practices in the Oxford RCGP RSC network on 11 May 2020 and having ≥1 year of health records in the network (*n* = 4 413 734). The network extracts pseudonymised data from primary health care electronic records of member practices and is recruited to be nationally representative (see Supplementary Figures S1 and S2 for details).^12^ Data include demographics, clinical conditions, medications, and laboratory results. The network also reports on mortality (Supplementary Figure S3).

### Study variables

The main outcome of interest was all-cause mortality, obtained from primary care CMRs over the entire period of analysis: weeks 2–20 of 2019 and 2020 (7 January–19 May 2019, and 6 January–18 May 2020). Mortality data were obtained from a combination of coded data entered into the clinical record to indicate that the patient had died, and examination of patients who had been removed from the practice list by the national demographic service, which flags those who have died. Where available, the coded date of death was preferentially used.

The primary variables of interest included living in communal dwellings, SARS-CoV-2 exposure, socioeconomic and ethnic inequalities, and also learning disabilities. People were grouped into the same household based on having identical addresses, and households were divided into those with 1, 2–4, 5–8, and ≥9 residents. Residences with ≥9 people were described as communal establishments. This matching was done programmatically at data extraction without researchers having access to personal addresses. SARS-CoV-2 status was classified at four levels:

- definite case — supported by a positive virological test result;
- probable case — based on positive clinical code in the absence of a test;
- possible case — a code suggested testing for SARS-CoV-2 (but no result), clinical suspicion, or contact; and
- not a case — people with negative test results (see Supplementary Tables S1a and S1b for coding lists).

The SARS-CoV-2 status algorithm worked hierarchically, so if probable or possible cases subsequently had a negative test they were reclassified as not a case.

Other variables included:

- age;
- sex;
- socioeconomic status using the index of multiple deprivation (IMD), based on lower super output area (LSOA) — a geographical subunit with a minimum population of 1000 — divided into quintiles;^13^
- ethnicity divided into white, Asian, black, mixed, and others;^14^ and
- household size; determined using a pseudonymised household key based on identical address, this has been used in other studies.^10^^,^^15^^,^^16^
- smoking status (comparing current, ex- and non-smokers);
- obesity (using the World Health Organization categorisation of overweight [body mass index {BMI} = 25–29 kg/m^2^]; obese [BMI = 30–34 kg/m^2^]; and severely obese [BMI≥35 kg/m^2^]; and
- population density (based on ONS locality data).^17^ The highest population density was in ‘conurbations’, medium levels in ‘city and town’, and lowest density in ‘rural areas’.

The following disease groups or clinical risk groups that might be associated with adverse outcomes were added in case codes as surrogates for SARS-CoV-2 exposure: upper and lower respiratory infections (URTI and LRTI, respectively), Type 1 and type 2 diabetes mellitus, hypertension, chronic kidney disease (CKD) defined as stage 3–5,^18^ heart disease (including myocardial infarction, other forms of coronary artery disease, and heart failure), chronic respiratory disease (asthma, chronic obstructive pulmonary disease, bronchiectasis, and other chronic lung conditions), people undergoing treatment for cancer or who may be immunocompromised due to taking medications for inflammatory conditions, and people with learning disability (Table 1).

**Table 1.** Oxford RCGP RSC cohort with known SARS-CoV-2 status

**Variable**		***n* (N = 56 628)**	**%**
SARS-CoV-2 status	Not Detected	6786	12.0
	Possible	42 390	74.9
	Probable	2710	4.8
	Definite	4742	8.4

Death	No	54 518	96.3
	Yes	2110	3.7

Upper respiratory infections	No	48 691	86.0
	Yes	7937	14.0

Lower respiratory infections	No	47 970	84.7
	Yes	8658	15.3

Age band, years	≤65	39 537	69.8
	65–74	6381	11.3
	≥75	10 710	18.9

Sex	Female	33 578	59.3
	Male	23 050	40.7

Household Size	1	13 176	23.3
	2 to 4	32 518	57.4
	5 to 8	6143	10.8
	≥9	3583	6.3
	Missing	1208	2.1

Population density	Conurbation	12 582	22.2
	City & Town	31 951	56.4
	Rural	12 095	21.4

Index of multiple deprivation quintile	Most deprived, 1	9939	17.6
	2	11 852	20.9
	3	12 031	21.2
	4	11 286	19.9
	Least deprived, 5	11 520	20.3

Ethnicity	White	37 983	67.1
	Asian	3439	6.1
	Black	1495	2.6
	Mixed, Other	1232	2.2
	Missing	12 479	22

Body mass index band	Normal weight	19 167	33.8
	Overweight	15 504	27.4
	Obese	12 215	21.6
	Severely obese	2786	4.9
	Missing	6956	12.3

Smoking status	Non-smoker	7716	13.6
	Active	25 220	44.5
	Ex-smoker	17 962	31.7
	Missing	5730	10.1

Diabetes type	None	49 325	87.1
	Type-1	359	0.6
	Type-2	6944	12.3

Hypertension	No	41 263	72.9
	Yes	15 365	27.1

Chronic kidney disease	No	53 055	93.7
	Yes	3573	6.3

Chronic heart disease	No	48 417	85.5
	Yes	8211	14.5

Chronic respiratory disease	No	52 651	93.0
	Yes	3977	7.0

Malignancy, immunocompromised	No	48 846	86.3
	Yes	7782	13.7

Learning disability	No	55 951	98.8
	Yes	677	1.2

RCGP RSC = Oxford Royal College of General Practitioners Research and Surveillance Centre network.

### Statistical analysis

Trends in mortality for weeks 2–20 of 2020 were analysed using descriptive statistics, and excess deaths due to COVID-19 modelled using a relative survival model.^19^^,^^20^ In a relative survival model, the observed mortality rate in a cohort is compared to the age and sex specific mortality rates from a reference population. Assuming that the reference population mortality (that is, the background mortality from 2019) closely approximates what would have occurred in the absence of the disease, the difference between the observed and expected mortality will be close to cause-specific mortality.

The date of death as recorded in the primary care record was used. Each model was adjusted for age, sex, and household size. To ensure better model fit, age was examined as a continuous variable in the relative survival model, using a cubic polynomial term. The latest UK life expectancy tables were downloaded from the ONS via the Human Mortality Database maintained by the Max Planck Institute for Demographic Research.^21^ The life expectancy tables were imported as rate tables into the relative survival model. Current SARS-CoV-2 exposed (in weeks 2–20 of 2020) survival data were compared with these rates, enabling exposed survival to be measured relative to the counterfactual, exposure-free expected survival.^22^ Relative hazard ratios (RHR) and 95% confidence intervals (CI) were determined.

In patients with available SARS-CoV-2 status data, a multivariable logistic-regression model was fitted to examine the effect of age, sex, SARS-CoV-2 infection status, and household size on mortality. Further fully adjusted models examined these variables along with ethnicity, patient demographics (socioeconomic status and population density), smoking status, and underlying health conditions, including learning difficulties. Multiple imputation by the chained equations method was used (using all model covariates in the missingness model, including outcome but with no auxiliary variables) to impute missing data, imputing five datasets using predictive mean matching.^23^ Each dataset was inverse probability weighted using an iterative proportional fitting algorithm to match the marginal covariate distributions of each imputed dataset to the full RCGP RSC population margins.^24^ Outputs were employed in final multivariable, weighted logistic regressions.

Finally, each of the regression coefficient estimates, together with robust sandwich variance estimators, were pooled using Rubin’s rules.^25^ All analyses were undertaken using R (version 3.5.3). A complete cases analysis was conducted as a sensitivity analysis. Both models are reported using ORs with 95% CIs.

## RESULTS

### Mortality in England during the first SARS-CoV-2 peak

**Figure 1.**
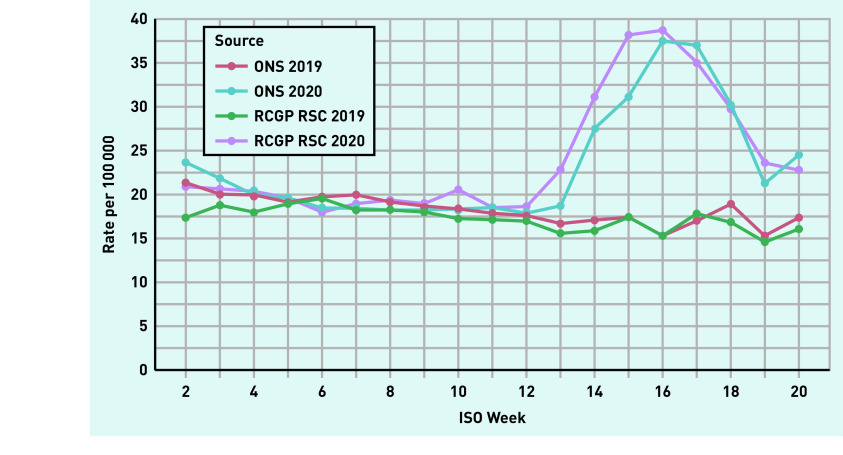
**Mortality per 100 000 across ISO Weeks 2–20 of 2019 and 2020 from sentinel network (RCGP RSC) and ONS ISO = International Standards Organization. ONS = Office of National Statistics. RCGP RSC = Oxford Royal College of General Practitioners Research and Surveillance Centre network. For tabulated mortality rates see Supplementary Table S4.**

The incidence of mortality during the first wave of SARS-CoV-2 peaked in week 16 (Figure 1) and rates observed in the RCGP RSC were very similar to national rates. There was excess mortality in weeks 14–20 of 2020 compared to the same period in 2019. Data on trends of SARS-CoV-2 infections show that the rate peaked slightly earlier in week 15, with the curve flattening between weeks 15 and 16 (see Supplementary Figure S4 for details).

### Excess mortality in 2020 compared to 2019

Compared with 2019, the relative survival model showed males to have a 13.2% greater risk of mortality than females (RHR = 1.13, 95% CI = 1.1 to 1.17, *P*<0.0001) but older people had a 1.8% reduced risk of mortality (RHR = 0.982, 95% CI = 0.978 to 0.982, *P*<0.0001).

Large household size, including communal establishments was strongly associated with a higher hazard of mortality in 2020 compared to 2019 (Table 2). Compared with single occupancy, households of 2–4 had a lower RHR, rising to 47% relative hazard in households of 5–8, then increasing fivefold for dwellings of ≥9 occupants (RHR = 5.1, 95% CI = 4.87 to 5.31, *P*<0.0001) (Table 2).

**Table 2.** Estimated relative hazard rates in the RCGP RSC population

	**RHR**	**95 % CI**	***P-*value**	**Coefficient**
**Sex**(ref category: female)	1.132	1.100 to 1.170	<0.0001	0.124

**Age** (continuous)	0.980	0.978 to 0.982	<0.0001	−0.020

**Household size** (ref category: 1)				
2–4	0.785	0.756 to 0.815	<0.0001	−0.242
5–8	1.465	1.359 to 1.579	<0.0001	0.382
≥9	5.082	4.869 to 5.305	<0.0001	1.626

CI = confidence interval. RCGP RSC = Oxford Royal College of General Practitioners Research and Surveillance Centre network. RHR = relative hazard ratio.

### Association of SARS-CoV-2 status with mortality

The cohort with known SARS-CoV-2 status (*n* = 56 628) were divided into definite cases confirmed by laboratory test (8.4%, *n* = 4742), probable cases with a firm clinical diagnosis (4.8%, *n* = 2710), possible infections (74.9%, *n* = 42 390), and those with a negative test (12.0%, *n* = 6786). For details of SARS-CoV-2 status across all study variables see Supplementary Table 2.

A total of 2110 (3.7%) individuals with recorded SARS-CoV-2 status died during the study period. The crude and adjusted rates of mortality were highest in those of male sex, aged ≥75 years, of probable or definite SARS-CoV-2 status, and living in households of single occupancy and ≥9 people (Table 3).

**Table 3.** Mortality, unadjusted and adjusted for sex, age, SARS-CoV-2 status, and household size in people with known SARS-CoV-2 status

	**Category**	**Deaths**	**N**	**Unadjusted Mortality, % (95 % CI)**	**Adjusted Mortality, % (95 % CI)**
**Sex**	Female	985	33 578	2.93 (2.76 to 3.12)	2.96 (2.88 to 3.05)
	Male	1125	23 050	4.88 (4.61 to 5.17)	5.09 (4.95 to 5.24)

**Age band, years**	≤64	212	39 537	0.54 (0.467 to 0.613)	0.42 (0.32 to 0.52)
	65–74	320	6381	5.01 (4.49 to 5.58)	5.02 (4.84 to 5.2)
	≥75	1578	10 710	14.73 (14.1 to 15.4)	15.71 (15.4 to 16.0)

**SARS-CoV-2 status**	Not detected	121	6786	1.78 (1.48 to 2.13)	1.88 (1.8 to 1.96)
	Possible	901	42 390	2.13 (1.99 to 2.27)	1.76 (1.72 to 1.79)
	Probable	399	2710	14.72 (13.4 to 16.1)	16.2 (15.4 to 16.9)
	Definite	689	4742	14.53 (13.5 to 15.6)	18.1 (17.6 to 18.7)

**Household size**	1 person	616	13 176	4.68 (4.32 to 5.05)	4.64 (4.48 to 4.80)
	2–4 persons	620	32 518	1.91 (1.76 to 2.06)	1.91 (1.85 to 1.96)
	5–8 persons	121	6143	1.97 (1.64 to 2.35)	1.76 (1.62 to 1.90)
	≥9 persons	734	3583	20.49 (19.17 to 21.84)	22.26 (21.60 to 22.93)

CI = confidence interval.

In multivariable analyses (Table 4), the odds of mortality were higher in those with probable and definite SARS-CoV-2 cases. Those of male sex (OR = 1.77, 95% CI = 1.37 to 2.03, *P*<0.0001), aged 65–74 years (OR 7.93, 95% CI = 6.42 to 9.77, *P*<0.0001), and aged ≥75 years were all associated with higher odds of mortality (OR = 18.71, 95% CI = 15.17 to 23.08, *P*<0.0001).

**Table 4.** Multivariable adjusted odds ratios for all-cause mortality in the RCGP RSC cohort with known SARS-COV-2 status

**Variable/category**	**OR**	**95% CI**	***P-*value**
**SARS-COV-2 status** (ref: negative test)			
Possible	1.5401	1.1649 to 2.0362	0.0024
Probable	9.6763	7.1185 to 13.1533	<0.0001
Definite	8.9032	6.6730 to 11.8788	<0.0001

**URTI** (ref: no URTI)	0.5522	0.2781 to 1.0963	0.0896

**LRTI** (ref: no LRTI)	1.2389	0.8367 to 1.8345	0.2847

**Age band, years** (ref: <65)			
65–74	7.9265	6.4285 to 9.7735	<0.0001
≥75	18.7132	15.1709 to 23.0826	<0.0001

**Male sex** (ref: female)	1.7665	1.3735 to 2.0340	<0.0001

**Household size** (ref: single occupancy)			
2–4 people	0.8151	0.6958 to 0.9549	0.0114
5–8 people	1.1629	0.9027 to 1.4983	0.2429
≥9	2.8045	2.2784 to 3.4522	<0.0001

**Population density** (ref: city and town)			
Conurbation	1.2887	1.0991 to 1.5109	0.0018
Rural	0.9207	0.7577 to 1.1187	0.4058

**Deprivation IMD quintile** (ref: 1 — most deprived)			
2	0.9872	0.8082 to 1.2058	0.8992
3	0.9234	0.7364 to 1.1579	0.4900
4	0.9246	0.7501 to 1.1398	0.4628
5 (least deprived)	1.0516	0.8435 to 1.3111	0.6545

**Ethnicity** (ref: white)			
Asian	1.2842	0.9613 to 1.7154	0.0904
Black	1.8424	1.3342 to 2.5440	0.0002
Mixed, Other	1.3162	0.8616 to 2.0105	0.2037

**BMI band** (ref: normal weight)			
Overweight	0.7966	0.6819 to 0.9306	0.0041
Obese	0.8547	0.7073 to 1.0328	0.1039
Severely obese	1.5323	1.0061 to 2.3335	0.0468

**Smoking status** (ref: non-smoker)			
Active	0.7925	0.5416 to 1.0894	0.0910
Ex-smoker	0.5700	0.4512 to 0.7202	0.0001

**Long-term conditions:** (ref: absence of condition)			
Type 1 diabetes	0.9607	0.2856 to 3.2313	0.9483
Type 2 diabetes	1.1982	0.9968 to 1.4403	0.0542
Hypertension	1.0897	0.9383 to 1.2654	0.2603
Chronic kidney disease	1.4131	1.1618 to 1.7187	0.0005
Chronic heart disease	1.1814	1.0046 to 1.3893	0.0438
Chronic respiratory	1.2986	1.0173 to 1.6575	0.0359
Cancer or immunocompromised	1.2972	1.0776 to 1.5616	0.0060
Learning disability	1.9682	1.2186 to 3.1788	0.0056

BMI = body mass index. CI = confidence interval. IMD = index of multiple deprivation. LRTI = lower respiratory infections. OR = odds ratio. ref = reference category. RCGP RSC = Oxford Royal College of General Practitioners Research and Surveillance Centre network. URTI = upper respiratory infections.

Compared with single occupancy, households with ≥9 occupants (including communal dwellings) were associated with higher mortality (OR = 2.8, 95% CI = 2.28 to 3.45, *P*<0.0001). Conurbations had a higher odds of mortality compared with city and town, with no difference in rural areas. Compared with white ethnicity, black ethnicity was associated with increased mortality (OR = 1.84, 95% CI = 1.33 to 2.54, *P* = 0.0002).

No change was seen in association of mortality with socioeconomic status, measured using IMD quintile. Ex-smokers had lower odds of mortality (OR = 0.57, 95% CI = 0.45 to 0.72, *P* = 0.0001), with no reduction of odds in current smokers. People diagnosed with chronic diseases and with learning disabilities (OR = 1.97, 95% CI = 1.22 to 3.18, *P* = 0.0056) had a higher odds of mortality, with the exception of diabetes and hypertension.

Analyses using complete cases only produced very similar findings (see Supplementary Table S3 for details). The only differences were that a clinical diagnosis of URTI was associated with a lower OR of mortality and that of a LRTI with a higher OR of mortality compared with not having these conditions (OR = 0.63, 95% CI = 0.50 to 0.79, *P* = 0.0001; OR = 1.35, 95% CI = 1.17 to 1.56, *P* = 0.0001). Additionally, there was a positive association between a diagnosis of type 2 diabetes and mortality (OR = 1.15, 95% CI = 1.01 to 1.32, *P* = 0.034).

## DISCUSSION

### Summary

These data show an excess in mortality in England associated with peak in SARS-CoV-2 virus circulation. Mortality rates per 100 000 population doubled over a 3-week period and then declined over the following 3 weeks to slightly above those seen in the previous year. There was an increased mortality in males and larger households, with establishments with ≥9 occupants having a fivefold increased risk of mortality.

This nested study of people with known SARS-CoV-2 status were found to have similar results to the all cause mortality study over the same period.^26^^,^^27^ Definite and probable cases also had a tenfold stronger association with mortality than those with a negative test. Population density, black ethnicity, and most long-term conditions were already known to be associated with increased odds of mortality. This study also added people with learning disability to the list of groups who have been more vulnerable to mortality associated with SARS-CoV-2 infection. These data highlight vulnerable groups that have experienced excess mortality during the first wave of SARS-CoV-2. Importantly, this excess mortality remains even after adjustment for SARS-CoV-2 status within the model. Measures should be taken to ensure that these groups are protected should a second wave occur in the future.

### Strengths and limitations

The strengths of this study are that it builds on >50 years’ experience of processing routine data for influenza and other infectious diseases in the English sentinel system.^7^ Selection bias has been adjusted for using a substantial number of demographic and health-related factors.^28^ It is noted that the findings in this population based study — in particular with respect to male sex, black ethnicity, household size, and comorbidities — are compatible with those with the known SARS-COV-2 status cohort used in this study.^26^ A similar pattern in excess mortality was found using a different approach.^27^

The use of clinical diagnostic codes (used to define the probable cases) is open to criticism. However, the authors feel their use is justified based on the similarity in unadjusted and adjusted mortality (Table 3) and the year-on-year experience of the utility of primary care diagnostic data correlating with circulating viral illness, most notably influenza.

Notwithstanding attempts to adjust for selection bias, the authors do not believe that ex-smokers have any real protective effect; there may have been another mechanism, such as a lower threshold for presentation or more cough. Current smoking status has been shown to be associated with adverse outcomes in other studies and was not significant in the present analyses.^29^ These mortality data are derived from the primary care providers CMR system, either where recorded by the practice or through linkage to a central registry. There may be some more immediacy in these mortality data, which often reflect date of death, as compared with ONS which records the date of registration of death.

The SARS-CoV-2 known status cohort of this study is likely to have had more severe disease. There was little testing available at the start of the pandemic, and testing was largely restricted to those who were symptomatic within the surveillance system and to those who attended hospital. The initial focus within early testing was on those with possible travel-related exposure, which may have introduced its own bias, although these numbers were small. After this initial period, testing was focused on those with specific symptoms or severe disease. Testing is now much more widely available, but many of these test results do not find their way back into GP clinical records.

### Comparison with existing literature

This study’s findings about ethnicity are compatible with those from PHE and ONS in that they also report increased mortality in people of black ethnicity.^30^^,^^31^ However, these larger national samples also showed other groups: Bangladeshi and Pakistani, Indian, and mixed ethnicities had a higher mortality; and additionally an association with obesity. The PHE report also shows a link with deprivation, also highlighting the association of care homes with increased mortality. It is likely that this study’s sample was underpowered for these other groups, although it is possible that other variables, such as household size and population density might account for these differences.

There were significant amounts of missing data for both ethnicity and BMI (Table 1). It has been challenging to identify predictive symptoms for SARS-CoV-2 infection, and the lack of association with URTI and LRTI found in the main model fits with this.^32^ The association of chronic disease with adverse outcomes has also been reported, though other reports have included diabetes.^33^^–^^35^

The association of care homes has also been reported, including case-fatality rates as high as 32%. However, the association with larger households, but not intermediate size dwelling (5–8 persons) is new.^36^

### Implications for research and practice

A key challenge during the first wave of SARS-CoV-2 was identifying the groups within the general population who are most vulnerable. In this analysis, vulnerability appeared to include living in communal establishments, such as, care homes and areas of higher population density. There was also evidence that people living alone were at increased risk than those living in intermediate or smaller family-sized dwellings.

Plans for any second wave may need to take account of these factors: considering control of movements into and out of care homes and other multi-occupancy dwellings, as well as infection prevention and control measures within communal establishments, and titrating the intensity of public health measures to conurbation size and population density. In light of these findings, there may be the need to move from a national response to more nuanced regional or local responses to adjust for the local level of risk informed by national SARS-CoV-2 surveillance.

Further research is needed to better understand pathways of care during this first wave of the pandemic, including acute admission to hospital as an outcome, once these data are available. It should also be explored whether any failure to manage other conditions or provide care, contributed to the overall increased mortality across this period.
